# Angled multimode interferometer for bidirectional wavelength division (de)multiplexing

**DOI:** 10.1098/rsos.150270

**Published:** 2015-10-21

**Authors:** Y. Hu, D. J. Thomson, A. Z. Khokhar, S. Stanković, C. J. Mitchell, F. Y. Gardes, J. Soler Penades, G. Z. Mashanovich, G. T. Reed

**Affiliations:** Optoelectronics Research Centre, University of Southampton, Southampton SO17 1BJ, UK

**Keywords:** silicon photonics, infrared, wavelength division (de)multiplexer

## Abstract

We have demonstrated a bidirectional wavelength division (de)multiplexer (WDM) on the silicon-on-insulator platform using two 4-channel angled multimode interferometers (AMMIs) sharing the same multimode interference waveguide. An excellent match of the peak transmission wavelength of each channel between the two AMMIs was achieved. The input and output access waveguides were arranged in a configuration such that the propagation of light of one AMMI in the multimode interference waveguide suffered minimal perturbation by the input and output waveguides of the other AMMI. This type of device is ideal for the WDM system for datacom or telecom applications, e.g. an integrated optical transceiver, where the transmission wavelengths are required to match with the receiving wavelengths. The device also benefits from simple fabrication (as only a single lithography and etching step is required), improved convenience for the transceiver layout design, a reduction in tuning power and circuitry and efficient use of layout space. A low insertion loss of 3–4 dB, and low crosstalk of −15 to −20 dB, was achieved.

## 1. Introduction

**Figure 1.**
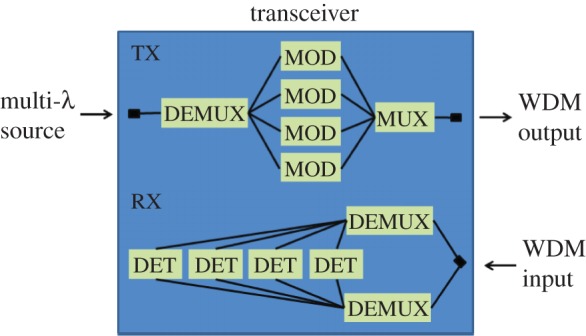
Diagram of the configuration of a silicon photonics transceiver featuring a multi-wavelength off-chip light source coupled to the chip on a single fibre and a polarization diversity scheme on the receiver side. DET, detector; MOD, modulator.

Wavelength division (de)multiplexers (WDMs) are key components for integrated optical transceivers. A variety of WDM structures have been developed [1–3] for integration into those transceivers. In a typical integrated optical transceiver, there are two wavelength matching WDMs, where one is for wavelength multiplexing (MUX) in the transmitter part and the other is for wavelength demultiplexing (DEMUX) in the receiver part. In a silicon photonics solution, a further DEMUX may be required to enable multi-wavelength light from an external source to be coupled to the chip via a single fibre, with the different wavelengths separated on-chip. This route may be preferred due to the difficulties brought about by forming a light source on the silicon chip. One further DEMUX may also then be required if a polarization diversity scheme is used in the receiver side of the transceiver. The final transceiver could therefore be as shown in figure 1, which contains up to four MUX/DEMUX structures. A highly uniform wafer platform and precise engineering are usually required to achieve a good match of these WDMs’ transmission wavelengths. This requirement is more stringent in a high-index-contrast platform, e.g. the silicon-on-insulator (SOI) platform, hence the spectral response of a WDM on the SOI platform is usually very sensitive to the dimensional error in fabrication; typically, this sensitivity is at a scale of 1 nm shift of transmission wavelength with respect to 1 nm error in waveguide’s dimension [4]. Recently, we demonstrated a WDM structure based on dispersive self-imaging in multimode waveguides—the angled multimode waveguide (AMMI). It has the distinct advantage of low sensitivity to fabrication variations and ease of fabrication compared with other WDM structures like arrayed waveguide gratings (AWGs) and Echelle gratings, as well as low insertion loss. The main disadvantage is a comparatively low channel count compared with AWGs so it is more suited to coarse wavelength division multiplexing. It was first demonstrated as a 4-channel device [5] and was recently applied to an 8-channel interleaved device [6]. Despite this improvement in sensitivity to fabrication variations, a 1-nm shift in the silicon layer thickness will still result in a shift in spectral response of 500 pm. It is, therefore, difficult to ensure that the MUX and DEMUX structures on the same chip are well aligned, and in reality a tuning mechanism would be required to tune each structure individually.

In this paper, we introduce a bidirectional AMMI structure on the SOI platform, which comprises two 4-channel AMMIs sharing the same multimode interference waveguide (MW). There are several advantages gained through using this structure rather than two separate devices as previously demonstrated for example in AWGs [7,8]. Firstly, the spectral response of the two AMMIs will theoretically be identical because the wavelength of the light which is transmitted from each of the output ports is determined by the properties of the MW which in this case is shared by the two structures. Secondly, a single-tuning element and control circuit can be applied to the MW which will shift the spectral response of the AMMI in either direction identically. Tuning is required to counteract fabrication variations and global temperature changes. A halving of the tuning power consumption and the control circuitry required can, therefore, be gained. Thirdly, a large amount of footprint is saved as half the number of bulky (DE)MUX structures are required. On the other hand, the additional input and output ports on the MW can interact with the propagating light of the first device degrading its performance. Further considerations are therefore required in the design of this device over that of the single AMMI in the positioning and size of the input waveguide (IW) and output waveguide (OW). Herein, details of the device design are presented together with theoretical and experimental analysis of the device. It is shown that an almost perfect match of the two AMMIs’ transmission wavelengths was achieved, demonstrating its potential as a practical solution to be employed in an integrated optical WDM transceiver. The performance in terms of insertion loss and crosstalk is slightly degraded from a single device, resulting in values of less than 4 dB and less than −18 dB, respectively. These values can be improved through further design optimization.

## 2. Design of bidirectional AMMI

**Figure 2.**
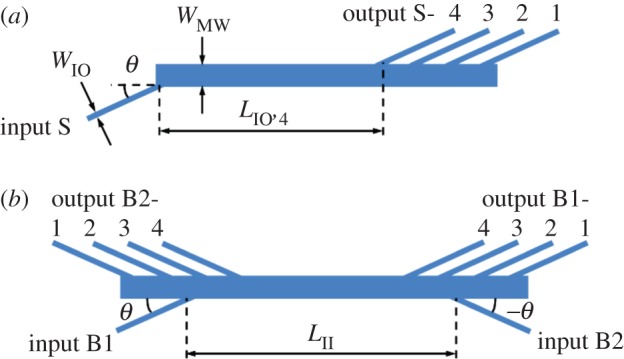
Design of (*a*) the single 4-channel AMMI and (*b*) the bidirectional AMMI.

The design starts from a single 4-channel AMMI, as shown in figure 2*a*. The single 4-channel AMMI has a long straight MW aligned horizontally. An IW intersects one side of the MW at an angle of *θ* with respect to the horizontal axis of the MW. The four OWs intersect the other side of the MW at the same angle. The horizontal distance between the IW’s intersection point and the *j*th OW’s intersection point is *L*_IO,*j*_. As mentioned in our previous study, the transmission wavelength of the output channel S-*j* should satisfy the dispersive self-imaging condition:

2.1 $$\lambda_{j}=\frac{4n_{\mathrm{eff},\mathrm{MW}}\times W_{\mathrm{MW}}^{2}}{L_{\mathrm{IO},j}}\;(\;j=1,2,3,4),$$

where *n*_eff,MW_ is the effective refractive index of the fundamental mode in the MW, *W*_MW_ is the width of the MW. The width of the IWs and OWs, *W*_IO_, and their tilt angle, *θ*, are not used in equation (2.1), but they affect the quality of the AMMI’s spectral response, e.g. insertion loss and crosstalk.

Based on the single 4-channel AMMI, we are able to construct another AMMI using the same MW. As shown in figure 2*b*, the IW of the second AMMI intersects the MW on the opposite side to the OWs of the first AMMI at an angle −*θ*, so that the IWs of the two AMMIs are on the same side of MW and the horizontal distance between their intersecting points to the MW is *L*_II_. To achieve the same spectral response of the first AMMI, the second AMMI should be a symmetric structure with respect to the first one; hence, the OWs of the second AMMI should intersect the MW on the opposite side to the IW of the first AMMI at an angle −*θ*. In this configuration, the two AMMIs share the same MW used for dispersive self-imaging and a good match of peak transmission wavelengths of the two MMIs is expected regardless of fabrication error, temperature change, etc.

**Figure 3.**
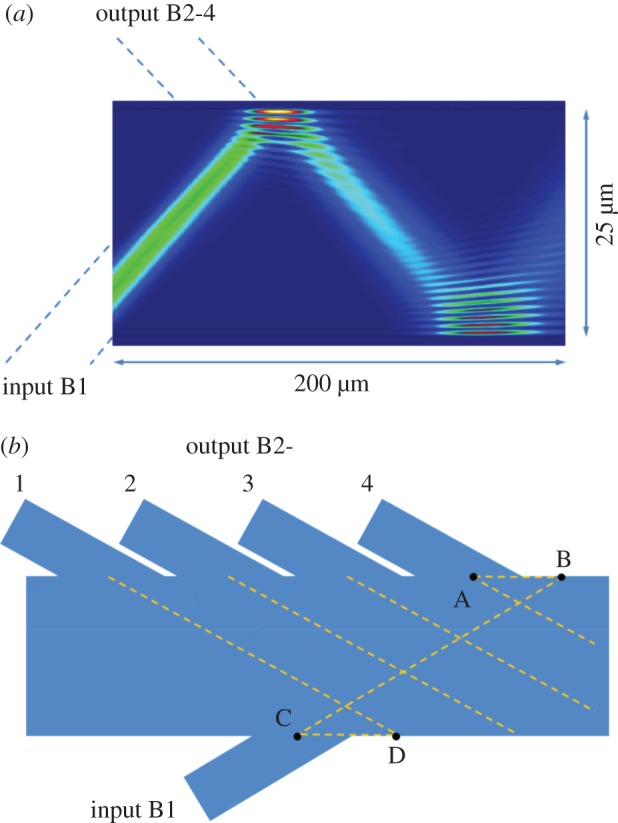
(*a*) Simulated field pattern at the first 200 μm length of the MW using the structural parameters in Hu *et al.* [6]. (*b*) The design of intersecting point of input B1 relative to the intersecting points of output B2.

Having the IW and OWs of the two AMMIs intersecting the same MW, light propagation in the MW of one AMMI could be perturbed by the IW and OWs of the other AMMI, and as a result, the spectral response of the bidirectional AMMI could have a higher insertion loss and a higher crosstalk compared with a single AMMI. The structural parameters should be designed to minimize this type of perturbation. Using the same structural parameters as in Hu *et al.* [6], we generated the field pattern in the MW with a commercial software package, FIMMPROP (http://www.photond.com/products/fimmprop.htm). As shown in figure 3*a*, light follows almost a straight line with little diffraction as it propagates from the IW’s intersecting point to the first reflection point on the other side of the MW. Considering the symmetry of field pattern in the self-imaging MW, light will also follow almost a straight line with little diffraction as it propagates from the last reflection point to intersection points of the OWs. Therefore, it is possible to use simple ray optics to analyse the propagation of the light in the MW at a distance less than 100 μm away from the IW and OWs. In the design shown in figure 3*b*, the input B1 points towards the other side of the MW at point B, which is to the right-hand side (RHS) of point A where the OW (B2-4) intersects the MIW; and the OW (B2-1) points towards the other side of the MW at point D, which is to the RHS of point C where the IW (B1) intersects the MW. The distances AB and CD are designed to balance the perturbation of output B2-4 to input B1 and that of input B1 to output B2-1; in our case, AB=CD=28 μm. Using the same SOI epitaxial layer structure as shown in Hu *et al.* [6], we found the optimized structural parameters for a single 4-channel AMMI, given by table 1. One may note that the IWs/OWs are designed to be wide enough (*W*_IO_=9.8 μm) so as to optimize the performance (minimum insertion loss and crosstalk) of a single 4-channel AMMI. It can be seen in figure 3*a* that the light from input B1 slightly overlaps with output B2-4 and, therefore, a reduction in *W*_IO_ could possibly suppress the light perturbation mentioned above while giving little degradation of insertion loss and crosstalk. This provides a route to optimize the performance of the device in the future.

**Table 1.** Designed structural parameters for a bidirectional AMMI.

*W*_IO_=9.8 μm, *θ*=16.6°, *W*_MW_=25 μm				
*L*_II_(μm)	*L*_IO,1_(μm)	*L*_IO,2_(μm)	*L*_IO,3_(μm)	*L*_IO,4_(μm)
4631	4700	4662	4625	4589

Light was coupled between the device and optical fibres using taper-grating units similar to those used in Hu *et al.* [5,6]. This unit is composed of a 450 nm-wide single-mode waveguide in connection with an adiabatic taper up to a 10 μm-wide waveguide. Ten 9.8 μm-wide input/output waveguides from the device were first connected to 450 nm-wide single-mode waveguides via adiabatic tapers which act as mode filters, and then to 10 taper-grating units. A separate structure incorporating two of the taper-grating units connected back-to-back was used for normalization.

## 3. Fabrication

The bidirectional AMMI together with a single 4-channel AMMI having the same design parameters as in table 1 were patterned on a high-resolution positive electron beam (e-beam) resist, ZEON ZEP520A, spun on a 6-inch SOI wafer with a silicon layer thickness of 400 nm and buried oxide (BOX) thickness of 2 μm. The wafer was then written by an e-beam lithography system, JEOL JBX-9300FS. For this particular work, this system produced a stable e-beam spot size of 50 nm and operated at an acceleration voltage of 100 kV. The wafer was patterned in stitching-error-free high-resolution mode within an area of 1×1 mm. When writing a large pattern, a stitching error of less than 20 nm was achieved with a positional accuracy of 1 nm. After a single e-beam writing step and resist development, the pattern was transferred to the wafer using inductively coupled plasma (ICP) etching, based on SF_6_ and C_4_F_8_ plasma. Finally, a 1 μm-thick SiO_2_ protecting layer was deposited on top of the whole wafer using a plasma-enhanced chemical vapour deposition (PECVD) at 350°C, before it was diced into chips for testing.

## 4. Results and discussion

**Figure 4.**
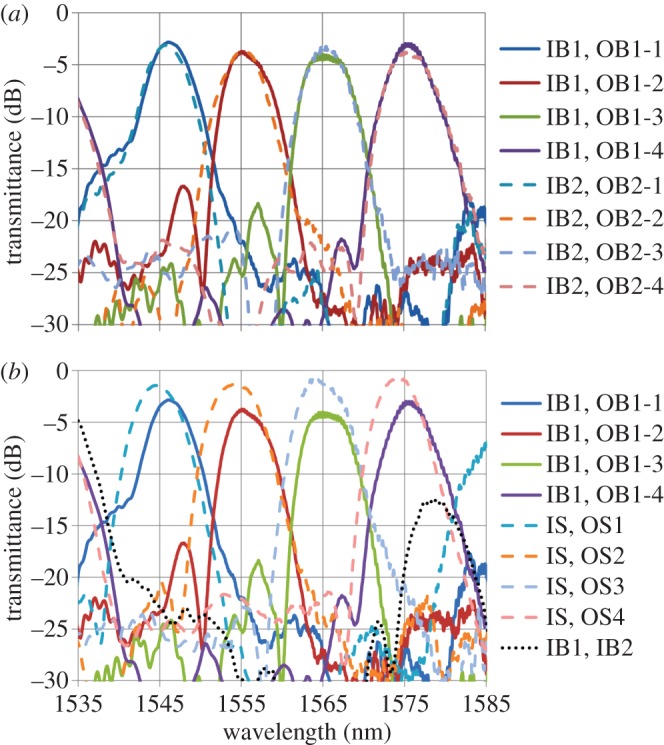
Spectral responses of fabricated devices. (*a*) The responses from input B1 to output B1 (solid lines) and from input B2 to output B2 (dashed lines) in the bidirectional AMMI. (*b*) The responses from input B1 to output B1 in the bidirectional AMMI (solid lines) and the single 4-channel AMMI (dashed lines) together with that from input B1 to input B2 in the bidirectional AMMI (dotted line).

Figure 4*a* shows the spectral responses of the fabricated bidirectional AMMI measured in both directions. One can see that an excellent match in the central wavelengths of the 4 channels from both directions is achieved with a mismatch between the corresponding channels of less than 0.1 nm. This figure of merit is of significant importance for achieving high-performance and stabilized operation in the optical transceiver. Any wavelength mismatch between the transmitted and received signals will increase the system’s insertion loss and crosstalk. In reality, different (DE)MUX structures in a transceiver will need to be tuned individually, firstly to achieve spectral alignment with each other, and secondly to counteract any shift in spectral response due to global temperature changes. Typically localized electrical heaters are used for this purpose in silicon photonics. By employing the bidirectional AMMI in the transceiver with negligible mismatch between transmitted and received wavelengths, the tuning mechanisms would only be needed to balance global temperature changes and furthermore because the MUX and DEMUX share the MW, a single tuning element can be used to shift both devices together. Reductions in power consumption and control circuitry are therefore achievable. Another advantage is the large reduction in footprint of the overall transceiver as only one MW is required for the two devices.

**Figure 5.**
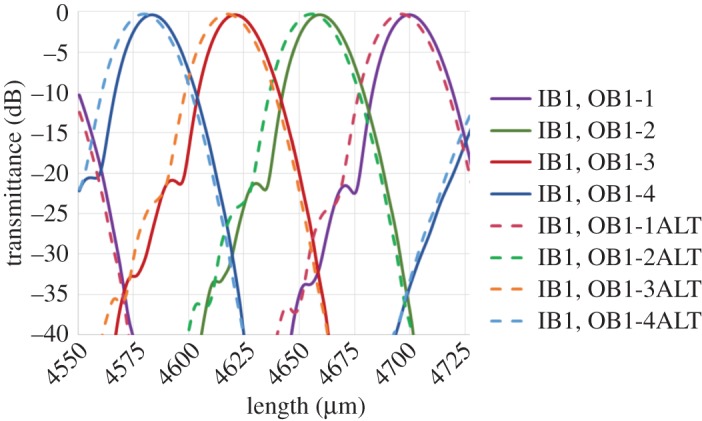
Simulated spectral responses: from input B1 to outputs B1-4 with nominal input/output waveguide width *W*_IO_ (solid lines), as well as from input B1 to outputs B1-4 with *W*_IO_ increased to 11 μm.

The insertion loss and the crosstalk achieved in the bidirectional AMMI are less than 4 dB and −18 dB, respectively. The spectral response of a fabricated single 4-channel AMMI placed closed to the bidirectional AMMI in the mask is shown in the dashed lines in figure 4*b*. The mismatch of the 4 channels’ central wavelengths between B1’s response in the bidirectional AMMI and the single AMMI is around 1 nm, which could result from the variation of device dimensions, e.g. the thickness of silicon layer or the width of MWs between the two devices. Using the bidirectional AMMI design, this type of mismatch has been reduced by one order of magnitude at the cost of an increase of insertion loss and crosstalk of about 2–3 dB and 3–5 dB, respectively. Another important consideration for the bidirectional AMMI is the cross-coupling between the two AMMIs sharing the same MW, which is detrimental to the transceiver system. The spectral response for the cross-coupling between input B1 and input B2 in the bidirectional AMMI is shown by the dotted line in figure 4*b*. Its central wavelength is slightly longer than channel 4 of the AMMI. From our analysis, this cross-coupling is due to the reflection of light from a point close to the intersecting point of output channel 4 (point B in figure 3*b*) to the input waveguide of the other AMMI (point A in figure 3*b*). One may consider keeping the distance of AB short or using wider output waveguides so that the reflection at point B cannot be disturbed by output channel 4 and the cross-coupling is suppressed. In consideration of all the issues mentioned previously, the design optimization of the bidirectional AMMI is the trade-off between improving spectral quality (to increase the distance of AB and CD and/or to decrease input/output waveguide width, *W*_IO_) and suppressing cross-coupling (to decrease the distance of AB and CD and/or to increase *W*_IO_). This effect can be seen in figure 5 where a simulation is shown for *W*_IO_ increased from 9.8 μm to 11 μm; a slight decrease in insertion loss can be appreciated, but at the same time this leads to an undesired increase in the crosstalk.

## 5. Conclusion

In conclusion, a bidirectional AMMI with two 4-channel AMMIs sharing the same MW was designed and demonstrated experimentally in the SOI platform. An insertion loss and crosstalk of less than 4 dB and less than −18 dB were achieved respectively. An excellent match of the central wavelengths of the two AMMIs is achieved. The bidirectional structure features advantages over using separate devices in precise channel alignment, the reduction of tuning power and control circuitry required, and the reduction of footprint. Such a device is attractive to the application of optical transceivers requiring close wavelength matching between transmitting and receiving channels. This type of device also benefits from the common advantages of a typical AMMI, such as low fabrication complexity, low insertion loss and crosstalk, etc. In the process of the design optimization, special attention was paid to maintaining low insertion loss and low crosstalk as well as suppression of cross-coupling between the two AMMIs.

## Data accessibility

The supporting data for the measured devices shown in this paper is available at http://dx.doi.org/10.5258/SOTON/381528.
